# 814. Impact of Transdermal Nicotine Replacement Therapy on Skin Graft Integrity Following Burn Injury

**DOI:** 10.1093/jbcr/irag033.253

**Published:** 2026-04-06

**Authors:** Savanna Faulconer, Kelsey G Glover, Rohit Mittal, Adria Johnson, Ashley B Hink, Steven A Kahn

**Affiliations:** Medical University of South Carolina, Charleston, SC; Medical University of South Carolina, Charleston, SC; South Carolina Burn Center at Medical University of South Carolina, Charleston, SC; Medical University of South Carolina, Charleston, SC; Medical University of South Carolina, Charleston, SC; Medical University of South Carolina, Charleston, SC

## Abstract

**Introduction:**

In burn patients, nicotine use is a known risk factor for poor wound healing and infection due to vasoconstrictive and pro-inflammatory effects. The use of transdermal nicotine patches in surgical patients in the perioperative period is controversial; but many large cohort studies suggest no major impact on outcomes. There is a paucity in the literature regarding burn patients and the use of nicotine replacement therapy (NRT), particularly regarding skin graft take. We hypothesize that the use of transdermal NRT does not significantly impact graft loss in burn patients with nicotine dependence.

**Methods:**

This single-center, retrospective study reviewed burn registry data over two years to identify current smokers with nicotine dependence who received NRT during admission. Demographics, infections, graft loss, and readmissions were compared between NRT and non-NRT patients with tobacco use disorder. Graft loss was defined as >25% loss with re-operation. Patients were only given NRT after an attending and the patient had a frank conversation about risks and benefits for patients unable to abstain without assistance.

**Results:**

Of 244 smokers, 58 received NRT and 186 did not. Groups were similar in demographics and injury characteristics: age (NRT: 46.1 yrs vs non-NRT: 44.6 yrs), TBSA (NRT: 5.4% vs non-NRT: 6.4%), and full-thickness burn size (1.4% vs 1.3%). The incidence of wound infection in the NRT vs non-NRT group was significantly higher 39.6% vs 21.5% (*p*=.009, RR 1.84); incidence of bacteremia was similar (5.1% vs 6.9%, *p*=.77). The readmission rate in the NRT vs the non-NRT group was significantly higher, 12.1% vs 2.6% (*p*=.009, RR 4.49). Among patients who received a skin graft, graft loss occurred in 4.8% (1/21) of the NRT group and in 3.6% (2/56) of the non-NRT group (*p*=1). Most infections were mild and successfully treated by increasing frequency of wound care, adding antimicrobial soaks, or antibiotics.

**Conclusions:**

Skin graft loss was less than 5% in this study and not increased with the use of NRT. However, NRT was associated with a 1.84-fold higher risk of wound infection affecting 39.6% of the group, along with a higher rate of readmission. NRT’s role in infection risk is unclear, as patients were nicotine dependent at the time of injury and this study cannot account for other confounding factors. Patients who received NRT likely had a selection bias for higher level of tobacco use, potentially making NRT a predictor of risk but not necessarily the cause of infection and readmission. Additional larger, prospective studies are needed to determine the true risk of infection and graft loss with NRT among patients with nicotine dependence.

**Applicability of Research to Practice:**

While the role of NRT in infection risk remains unclear, complications were manageable and there was no significant graft loss in NRT users, supporting its use after risk/benefit analysis for current smokers.

**Funding for the study:**

N/A.

